# Genetic dissection of heat-responsive physiological traits to improve adaptation and increase yield potential in soft winter wheat

**DOI:** 10.1186/s12864-020-6717-7

**Published:** 2020-04-20

**Authors:** Sumit Pradhan, Md Ali Babar, Guihua Bai, Jahangir Khan, Dipendra Shahi, Muhsin Avci, Jia Guo, Jordan McBreen, Senthold Asseng, Salvador Gezan, Byung-Kee Baik, Ann Blount, Stephen Harrison, Suraj Sapkota, Paul St. Amand, Sanju Kunwar

**Affiliations:** 1 Department of Agronomy, University of Florida, Gainesville, FL USA; 2 USDA-ARS, Manhattan, Kansas, USA; 3 Agricultural and Biological Engineering, University of Florida, Gainesville, FL USA; 4 School of Forest Resources and Conservation, University of Florida, Gainesville, FL USA; 5 USDA-ARS, Wooster, OH USA; 6 North Florida Research and Education Cente, Quincy, FL USA; 7 LSU AgCenter – SPESS, Baton Rouge, LA USA; 8 Institute of Plant Breeding, Genetics, and Genomics, University of Georgia, Athens, GA USA; 9 USDA-ARS, Manhattan, KS USA; 10 Department of Plant Pathology, University of Wisconsin-Madison, Madison, WI USA

**Keywords:** Single nucleotide polymorphisms_1_, Genotyping-by-sequencing_2_, Marker-trait associations_3_, Quantitative trait loci_4_, Genome-wide association study_5_, Physiological traits_6_, Marker-assisted breeding_7_

## Abstract

**Background:**

Climate change, including higher temperatures (HT) has a detrimental impact on wheat productivity and modeling studies predict more frequent heat waves in the future. Wheat growth can be impaired by high daytime and nighttime temperature at any developmental stage, especially during the grain filling stage. Leaf chlorophyll content, leaf greenness, cell membrane thermostability, and canopy temperature have been proposed as candidate traits to improve crop adaptation and yield potential of wheat under HT. Nonetheless, a significant gap exists in knowledge of genetic backgrounds associated with these physiological traits. Identifying genetic loci associated with these traits can facilitate physiological breeding for increased yield potential under high temperature stress condition in wheat.

**Results:**

We conducted genome-wide association study (GWAS) on a 236 elite soft wheat association mapping panel using 27,466 high quality single nucleotide polymorphism markers. The panel was phenotyped for three years in two locations where heat shock was common. GWAS identified 500 significant marker-trait associations (MTAs) (*p* ≤ 9.99 × 10^− 4^). Ten MTAs with pleiotropic effects detected on chromosomes 1D, 2B, 3A, 3B, 6A, 7B, and 7D are potentially important targets for selection. Five MTAs associated with physiological traits had pleiotropic effects on grain yield and yield-related traits. Seventy-five MTAs were consistently expressed over several environments indicating stability and more than half of these stable MTAs were found in genes encoding different types of proteins associated with heat stress.

**Conclusions:**

We identified 500 significant MTAs in soft winter wheat under HT stress. We found several stable loci across environments and pleiotropic markers controlling physiological and agronomic traits. After further validation, these MTAs can be used in marker-assisted selection and breeding to develop varieties with high stability for grain yield under high temperature.

## Background

Worldwide, wheat is grown on more than 218 million hectares of land and provides approximately 20% of dietary calorie needs [1]. Although, there has been a substantial increase in yield since the Green Revolution, the pace of increase in yield production is not predicted to match demand resulting from increase in human population and changing weather pattern [2, 3]. High temperature (HT) stress is one of the major consequences of climate change and poses a serious threat to wheat production [4]. Global temperature has increased by 0.5 °C in the twentieth century [5] and this warming trend is expected to continue up to 1.5–4.5 °C by the end of twenty-first century, resulting in elevated daytime maximum (HDT) and nighttime minimum temperatures. (HNT) [6]. Post-anthesis heat stress is very common in wheat growing areas and can cause large reductions in grain yield [7]. Although, some researchers suggest that HDT and HNT cause damage of a similar magnitude to winter wheat [8], others report a stronger negative impact on yield of HNT compared to HDT [9].

Genetic improvement of yield under HT via direct selection is hindered by the quantitative nature of grain yield and large genotype by environment interaction. Optimizing carbohydrate partitioning using traits such as spike fertility (SF), internode partitioning, and spike organ partitioning is essential to overcome sink limitations and increase harvest index (HI) [10, 11]. Although higher grain yield potential of wheat has been largely a result of increased HI under HT, increased biomass and crop adaptation are equally important. Physiological trait (PT) selection insures development of stress resilient genotypes with functioning metabolic activities including photosynthesis and respiration under HT [12]. Moreover, previous studies have reported strong correlations of these traits with grain yield. Therefore, PTs can serve as indirect selection tools to select superior genotypes from large numbers of breeding lines for stress environments and to compensate for a large genotype by environment interaction. This is important to wheat breeders since it can save substantial amounts of labor, time, and money and permits rapid screening of a large number of genotypes in relatively short time [13, 14]. Selection of desirable physiological trait (PTs) associated with heat adaptation and combinations is essential for future improvement of crops and provides opportunities for optimizing genetic yield gain. A model proposed to improve yield in wheat under heat stress includes partitioning of assimilates, radiation use efficiency (RUE), and light interception (LI) [15]. Some of the candidate PTs associated with these components are well documented as being heat adaptive traits, including higher leaf chlorophyll content measured as SPAD (soil-plant analyses development) value, intact leaf greenness measured as normalized difference vegetation index (NDVI), membrane thermostability (MT), and canopy temperature (CT). While these PTs are good candidates for improving heat tolerance and yield potential in wheat, limited knowledge on their genetic basis prevent full exploitation [11, 16]. Use of these indirect selection tools is limited in breeding programs due to their complex evaluation procedure [17]. Therefore, identifying novel genetic loci (QTLs) associated with PTs under heat stress and using them as selection tools can results in a cumulative genetic effect on yield, which is the basis of maker assisted physiological trait breeding [18].

Association mapping is a powerful approach that utilizes genetic diversity and historical recombination events to provide a high resolution map of trait-linked loci [19]. Although genome-wide association studies (GWAS) have been used to identify quantitative trait loci (QTL) in wheat for various simply inherited traits like disease resistance [20], currently, limited information are available for GWAS of complex PTs, particularly under HT. Recently, the International Wheat Genome Sequencing Consortium (IWGSC) published a full chromosome-anchored reference genome which allows more precise curation of marker trait associations (MTAs) identified by GWAS. In this study, GWAS was performed on 236 advanced soft red winter wheat accessions using 27,466 SNPs generated by GBS. The panel was phenotyped at two heat stress locations over three years. The objectives of this study were: i) to identify novel MTAs linked to NDVI, CT, SPAD and MT under HT and ii) to identify candidate genes for these MTAs and investigate their underlying function.

## Results

### Phenotypic analyses

There was significant genotypic variation (*P* < 0.001) for all measured traits (Additional file 3), as expected given the diverse genetic backgrounds of the SWAMP. Environments (growing years and locations) and their interaction were all significant (*P* < 0.05) determinants of phenotypic traits except for MT (Additional file 3). Trait means and a summary in response to each environment are provided (Table 1). All traits had moderate heritabilities, ranging from 60% for MT to 35% for CT (Table 1).

**Table 1:** Summary of adjusted means of physiological traits for the SWAMP

**Traits**	**Citra**		**Quincy**		**Combined**		***H***^***2***^
	Mean	Range	Mean	Range	Mean	Range	
**SPAD**	51.11	25.68–61.16	48.57	13.28–52.55	50.25	31.56–61.36	0.49
**MT**	56.72	22.27–77.21	59.47	23.68–61.95	57.63	26.29–74.26	0.60
**CT**	26.38	24.87–28.23	23.65	21.47–25.41	25.28	23.91–26.72	0.35
**NDVIa**	0.73	0.58–0.80	0.74	0.59–0.86	0.73	0.62–0.81	0.56
**NDVIg**	0.59	0.37–0.77	0.62	0.41–0.76	0.61	0.43–0.75	0.40

SPAD, soil-plant analyses development; MT, cell membrane thermostability (%); CT, canopy temperature (°C); NDVIa, normalized difference vegetation index at GS65; NDVIg, normalized difference vegetation index at grain filling

Pearson correlation coefficients (r) among PTs and their relationship with GY, GN, SF, HI, SHI and TGW were calculated using the combined dataset. SPAD was positively correlated with MT (0.31***), GY (0.50***), SF (0.25***), GN (0.30***), HI (0.46***), SHI (0.37***) and TGW (0.26***) (Additional file 4). Similarly, MT was positively correlated with NDVIa (0.22***), NDVIg (0.31***), GY (0.60***), SF (0.29***), GN (0.33***), HI (0.58***), SHI (0.44***) and TGW (0.40***). Pearson correlation coefficients results were further supported by principal components (PC) analysis that showed SPAD and MT were closely associated with GY, SF, GN, HI and SHI (Additional file 5). CT was negatively correlated with GY (− 0.18**), GN (− 0.17*), MT (− 0.25***), and SPAD (− 0.25***). NDVIa and NDVIg were positively correlated with GY, GN, HI and TGW (Additional file 4).

### Genetic data, LD decay, and population structure

Population structure analysis of the SWAMP was performed in our previous study using 27,466 high quality GBS-derived SNP markers (minor allele frequency; MAF > 0.05 and missing data < 20%) [10]. Briefly, these SNP markers were distributed throughout the A (9958, ~ 36%), B (9,968, ~ 36%) and D (6,954, ~ 25%) genomes. A total of 686 SNPs where found on unplaced scaffolds and thus were classified as unmapped SNPs. Chromosome 2B had the highest number of SNPs (1960) and chromosome 4D had the lowest (571). The population structure analysis grouped the 236 SWAMP lines into three genetic demes containing 49, 144, and 43 lines respectively (Additional file 6). PC analysis revealed substantial admixture among lines in the SWAMP, with the first and second PC explaining only 4.7 and 3.1% of the total genotypic variance, respectively (Additional file 6).

LD was computed using the “LDcorSV” package in R to determine the approximate marker density required for GWAS [10]. The LD decay below the line of critical value (r^2^ = 0.2) was estimated at 1182, 1920 and 2916 bp for ranges of 30,000, 40,000, and 50,000 bp, respectively, across the whole genome (Additional file 7). The magnitude of change in LD decay between a sample range of 30,000–50,000 bp was 1734 bp. Population structure was investigated to avoid false positive associations in GWAS (Additional file 6) [19].

### Marker-trait association

The GWAS identified novel MTAs for all measured PTs and explained a large portion of phenotypic variances from 5 to 23% (Additional file 8). The FarmCPU model with kinship and PC scores was used to identify MTAs for each trait using 27,466 GBS-derived SNPs. The SNP markers were uniformly distributed throughout the chromosomes of each genome (Additional file 8). GWAS was conducted on three datasets: BLUEC (Citra), BLUEQ (Quincy) and BLUEA (combined). We identified 500 significant MTAs for PTs distributed across 21 chromosomes (Additional file 8). The highest number of MTAs was detected in BLUEA (192) followed by BLUEQ (177) and BLUEC (131) (Table 2). The highest number of MTAs were identified in the B genome (225 MTAs), compared to A (139 MTAs) and D (136 MTAs) genomes. We identified 94 MTAs for SPAD across three datasets on chromosomes with phenotypic variance explained (PVE) ranging from 13 to 20% (Table 2, Additional file 8). For MT, 95 MTAs were identified with PVEs ranging from 5 to 15%. We detected the highest number of MTAs for CT (110) with PVEs ranging from 8 to 13%. For NDVIa, and NDVIg we detected 102 and 99 significant MTAs respectively with PVEs ranging from 5 to 23%. (Table 2, Additional file 8).

**Table 2:** Summary of significant marker–trait associations for physiological traits

**Traits**	**BLUEC**	**BLUEQ**	**BLUEA**	**Total**
**SPAD**	28	33	33	94
**MT**	25	35	35	95
**CT**	24	49	37	110
**NDVIa**	22	32	48	102
**NDVIg**	32	28	39	99
**Total**	131	177	192	500

SPAD, soil-plant analyses development; MT, cell membrane thermostability (%); CT, canopy temperature (°C); NDVIa, normalized difference vegetation index at GS65; NDVIg, normalized difference vegetation index at grain filling. BLUEC, BLUEs values derived across Citra; BLUEQ, BLUEs values derived across Quincy, and BLUEA: BLUEs values derived across all environment

Co-localized MTAs controlling multiple PTs were detected in the study. Ten pleiotropic SNP markers on chromosomes 1D, 2B, 3A, 3B, 5A, 6A, 7B, and 7D were detected across different environments (Table 3). Seven of them were associated with NDVIa and NDVIg indicating common MTAs for NDVI expressed during anthesis and grain filling period. SNP S7D_635578722 had a positive allelic effect for SPAD, NDVIa and NDVIg. SNP S2B_466014434 was associated with SPAD and MT and had a negative allelic effect on both traits. S3A_609909640 was associated with SPAD and CT and had a negative allelic effect on CT and a positive allelic effect on SPAD (Table 3). Interestingly, we detected five significant MTAs on chromosome 3A (S3A_12554694 and S3A_12554700), 5A (S5A_590056740), 6B (S6B_149148874), and 7D (S7D_18808932) for PTs, which were associated with GY and other yield related traits in our previous study [10].

**Table 3:** List of significant markers associated with multiple phenotypic traits (pleiotropy) in the SWAMP

**SNP**	**Trait**	**Dataset**	**-log10(*p*)**	**Effect**	**PVE**
**S1D_479711964**	NDVIa	BLUEA	5.26	0.00	0.20
	NDVIa	BLUEC	5.52	0.01	0.23
	NDVIg	BLUEA	3.40	0.01	0.16
**S2B_466014434**	MT	BLUEC	3.31	−4.85	0.06
	SPAD	BLUEA	3.83	−1.89	0.20
	SPAD	BLUEC	3.34	−1.91	0.16
**S3A_609909640**	CT	BLUEC	4.11	−0.20	0.11
	SPAD	BLUEQ	4.51	1.78	0.16
**S3B_785311769**	NDVIa	BLUEC	6.02	0.00	0.22
	NDVIg	BLUEA	3.01	0.01	0.13
	NDVIg	BLUEQ	3.34	0.01	0.07
**S5A_356222133**	NDVIa	BLUEQ	3.26	0.01	0.11
	NDVIg	BLUEQ	3.02	0.02	0.08
**S5A_356222163**	NDVIa	BLUEQ	3.26	0.01	0.11
	NDVIg	BLUEQ	3.02	0.02	0.08
**S5A_590056740**	NDVIa	BLUEA	5.79	−0.01	0.18
	NDVIg	BLUEA	3.10	−0.02	0.13
**S6A_39961388**	NDVIa	BLUEC	3.12	0.01	0.22
	NDVIg	BLUEA	3.66	0.02	0.17
**S7B_701649275**	NDVIa	BLUEA	5.82	0.00	0.20
	NDVIg	BLUEA	3.78	0.01	0.15
**S7D_635578722**	NDVIa	BLUEA	3.49	0.01	0.18
	NDVIa	BLUEQ	3.34	0.02	0.09
	NDVIg	BLUEA	3.15	0.03	0.15
	NDVIg	BLUEC	4.17	0.02	0.11
	SPAD	BLUEQ	3.41	−3.40	0.15

SPAD, soil-plant analyses development; MT, cell membrane thermostability (%); CT, canopy temperature (°C); NDVIa, normalized difference vegetation index at GS65; NDVIg, normalized difference vegetation index at grain filling. BLUEC, BLUEs values derived across Citra; BLUEQ, BLUEs values derived across Quincy, and BLUEA: BLUEs values derived across all environment

Seventy-five out of 500 MTAs were expressed in multiple environments and were considered as stable MTAs (Table 4). We identified 23 stable markers for SPAD on chromosomes 1B, 1D, 2B, 2D, 3B, 4A, 5B, 5D, 6B, 6D, 7A, and 7D with PVEs ranging from 13 to 19% (Table 4, Additional file 8). For MT, we identified 20 stable MTAs on chromosomes 1B, 1D, 2A, 2D, 3A, 3D, 5B, 5D, 6A, 6B, and 7B with PVEs ranging from 5 to 14%. Similarly, 17 stable MTAs were detected for CT with PVE ranging from 8 to 13%. For NDVIa and NDVIg, we identified 3 and 12 stable markers respectively that were unique to corresponding growth stages (Table 4).

**Table 4:** List of significant markers expressed in multiple environments (stable) in the SWAMP

**SPAD**	**MT**	**CT**	**NDVIa**	**NDVIg**
S1B_4335636^AC^	S1B_602752201^AQ^	S2B_693094464^AQ^	S2B_717098540^AC^	S3A_699988530^AQ^
S1B_204428462^AC^	S1B_602752209^AQ^	S2B_693094466^AQ^	S3A_108025984^AC^	S3A_699991338^AQ^
S1D_907114^AQ^	S1B_602752224^AQ^	S5B_601343966^AQ^	S6D_201817286^AC^	S3A_732890228^AQ^
S1D_907133^AQ^	S1B_602752226^AQ^	S5B_602833771^AQ^		S3A_736970882^AQ^
S2B_466014437^AC^	S1D_262475151^AC^	S5B_606014586^AQ^		S3A_737114441^AQ^
S2D_574118879^AQ^	S2A_70446757^AC^	S5B_607207678^AQ^		S2D_570960728^AQ^
S2D_11171031^AC^	S2D_602734684^AC^	S5B_607207704^AQ^		S3B_785311773^AQ^
S3B_792189571^AQ^	S2D_634968398^AQ^	S5B_608350950^AQ^		S5B_583295527^AC^
S4A_625244392^AC^	S3A_12554694^AQ^	S5B_610295429^AQ^		S1B_677572998^AQ^
S5B_592791824^AQ^	S3A_12554700^AQ^	S5B_617291841^AQ^		S7B_426882778^AQ^
S5D_220760001^AC^	S3B_509660072^AC^	S5B_621237427^AQ^		S2A_93482025^AQ^
S6B_131285725^AC^	S3D_590224603^ACQ^	S5B_622494564^AQ^		S3B_784466451^AQ^
S6B_150497646^AC^	S3D_590224620^AC^	S5B_622494601^AQ^		
S6B_462165779^AC^	S5B_487440465^AQ^	S5B_622494604^AQ^		
S6D_16178496^AC^	S5B_509105168^AQ^	S5B_643470598^AQ^		
S6D_16178499^AC^	S5B_509105191^AQ^	S6B_149148874^AC^		
S6D_16178505^AC^	S5D_184110184^AC^	S7B_265453929^ACQ^		
S7A_563391742^AQ^	S6A_58259479^AQ^			
S7A_565347529^AQ^	S6B_42215716^AQ^			
S7A_579648980^AQ^	S7B_464657928^AQ^			
S7A_644864716^AC^				
S7A_644864763^AC^				
S7D_37142233^AC^				

^A^Combined; ^C^Citra; ^Q^Quincy

SPAD, soil-plant analyses development; MT, cell membrane thermostability (%); CT, canopy temperature (°C); NDVIa, normalized difference vegetation index at GS65; NDVIg, normalized difference vegetation index at grain filling. BLUEC, BLUEs values derived across Citra; BLUEQ, BLUEs values derived across Quincy, and BLUEA: BLUEs values derived across all environment

### Gene annotation

Functional annotation of all stable MTAs was carried out using the IWGSC v1.0 reference genome sequence assembly. Forty-one out of 75 stable MTAs associated with PTs were anchored within functional genes. These MTAs had a wide range of functional annotations and are potential candidate genes for QTLs of interest (Table 5). Candidate genes were further investigated using past literatures to understand their possible functions. We discovered that these candidate genes encode different classes of proteins including F-box family proteins, RNA-binding proteins, disease resistance protein and protein kinase that have suggestive roles in response to biotic and abiotic stresses (Table 5). In addition, gene annotation was also carried out for all the significant MTAs (Fig. 2). Interestingly, we identified several MTAs (linked to different traits) in different chromosomes that had common genes with exact same annotation (Fig. 2 and Additional file 8).

**Table 5:** List of potential candidate genes and anchoring markers associated with physiological traits

**SNP**	**SNP**	**Dataset**	**-log10(*p*)**	**Effec**t	**PVE**	**Alleles**	**Gene-ID**	**Annotation**
S1B_4335636	SPAD	BLUEA	3.46	1.19	0.17	G/**A**	TraesCS1B01G007900	E3 ubiquitin-protein ligase ORTHRUS 2
		BLUEC	3.01	1.20	0.14			
S1D_907114	SPAD	BLUEA	3.65	1.74	0.18	G/**C**	TraesCS1D01G003300	SNF1-related protein kinase regulatory subunit beta-2
		BLUEQ	3.13	2.33	0.14			
S1D_907133	SPAD	BLUEA	3.65	1.74	0.18	G/**C**	TraesCS1D01G003300	SNF1-related protein kinase regulatory subunit beta-2
		BLUEQ	3.13	2.33	0.14			
S2D_574118879	SPAD	BLUEA	3.98	2.78	0.19	A/**G**	TraesCS2D01G469000	GDSL-like Lipase/Acylhydrolase superfamily protein
		BLUEQ	3.09	3.53	0.14			
S2D_11171031	SPAD	BLUEC	3.48	1.42	0.15	T/**C**	TraesCS2D01G026300	Glutamyl-tRNA (Gln) amidotransferase subunit A
		BLUEA	3.07	1.21	0.17			
S4A_625244392	SPAD	BLUEA	3.35	1.63	0.17	C/**T**	TraesCS4A01G346600	F-box protein
		BLUEC	3.22	1.75	0.14			
S5D_220760001	SPAD	BLUEC	3.34	1.64	0.14	G/**A**	TraesCS5D01G138700	disease resistance protein (TIR-NBS-LRR class) family
		BLUEA	3.18	1.46	0.17			
S6D_16178496	SPAD	BLUEA	3.59	−1.66	0.19	C/**T**	TraesCS6D01G038900	2-oxoglutarate (2OG) and Fe (II)-dependent oxygenase superfamily protein
		BLUEC	3.55	−1.81	0.16			
S6D_16178499	SPAD	BLUEA	3.59	−1.66	0.19	A/**T**	TraesCS6D01G038900	2-oxoglutarate (2OG) and Fe (II)-dependent oxygenase superfamily protein
		BLUEC	3.55	−1.81	0.16			
S6D_16178505	SPAD	BLUEA	3.59	−1.66	0.19	A/**C**	TraesCS6D01G038900	2-oxoglutarate (2OG) and Fe (II)-dependent oxygenase superfamily protein
		BLUEC	3.55	−1.81	0.16			
S6B_462165779	SPAD	BLUEA	4.15	1.34	0.18	C/**G**	TraesCS6B01G257900	Adenosine kinase-like protein
		BLUEC	3.19	1.27	0.14			
S7A_565347529	SPAD	BLUEA	4.54	1.38	0.19	T/G	TraesCS7A01G389100	TBC1 domain family member
		BLUEQ	4.38	1.98	0.15			
S7A_579648980	SPAD	BLUEQ	3.63	−1.78	0.15	C/**G**	TraesCS7A01G399700	Telomere repeat-binding factor like-protein
		BLUEA	3.48	−1.19	0.17			
S1D_262475151	MT	BLUEC	3.53	4.15	0.07	T/**C**	TraesCS1D01G190700	Heavy metal transport/detoxification superfamily protein
		BLUEA	3.52	4.14	0.10			
S3D_590224603	MT	BLUEA	4.89	4.60	0.12	T/**C**	TraesCS3D01G501200	Protein kinase
		BLUEC	4.80	4.57	0.09			
		BLUEQ	3.20	4.98	0.12			
S3D_590224620	MT	BLUEC	3.61	4.21	0.06	G/**A**	TraesCS3D01G501200	Protein kinase
		BLUEA	3.12	3.87	0.09			
S5B_487440465	MT	BLUEQ	3.36	4.36	0.11	A/**C**	TraesCS5B01G302900	Plant/T31B5–30 protein
		BLUEA	3.23	3.11	0.09			
S5B_509105168	MT	BLUEQ	4.25	−6.33	0.14	A/**G**	TraesCS5B01G325000	F-box family protein
		BLUEA	3.78	−4.32	0.11			
S5B_509105191	MT	BLUEQ	4.25	6.33	0.14	T/**C**	TraesCS5B01G325000	F-box family protein
		BLUEA	3.78	4.32	0.11			
S6B_42215716	MT	BLUEC	3.46	6.04	0.07	A/**G**	TraesCS6B01G063500	Peroxidase
		BLUEA	3.08	5.64	0.09			
S2B_693094464	CT	BLUEC	3.83	−0.24	0.10	G/**A**	TraesCS2B01G496300	BTB/POZ and MATH domain-containing protein 2
		BLUEA	3.10	−0.16	0.08			
S2B_693094466	CT	BLUEC	3.83	−0.24	0.10	G/**A**	TraesCS2B01G496300	BTB/POZ and MATH domain-containing protein 2
		BLUEA	3.10	−0.16	0.08			
S5B_610295429	CT	BLUEA	4.41	0.29	0.12	T/**G**	TraesCS5B01G436700	Lipid transfer protein
		BLUEQ	4.27	0.34	0.11			
S5B_608350950	CT	BLUEQ	4.85	−0.34	0.12	C/**T**	TraesCS5B01G433700	Maintenance of telomere capping protein 2
		BLUEA	3.19	−0.23	0.09			
S5B_621237427	CT	BLUEQ	4.37	−0.34	0.11	C/**T**	TraesCS5B01G448700	Mitochondrial transcription termination factor-like
		BLUEA	3.22	−0.24	0.10			
S5B_606014586	CT	BLUEQ	3.37	0.30	0.10	T/**C**	TraesCS5B01G431300	Peptidase M50 family protein
		BLUEA	3.03	0.23	0.10			
S5B_602833771	CT	BLUEA	3.70	−0.24	0.11	C/**T**	TraesCS5B01G426900	DNA topoisomerase
		BLUEQ	3.64	−0.28	0.10			
S5B_617291841	CT	BLUEA	4.79	−0.30	0.13	A/**G**	TraesCS5B01G445300	Endo-1,4-beta-xylanase
		BLUEQ	3.51	−0.30	0.10			
S5B_643470598	CT	BLUEA	3.16	0.18	0.10	G/**A**	TraesCS5B01G470200	Protein phosphatase 2C
		BLUEQ	3.07	0.22	0.08			
S5B_601343966	CT	BLUEA	3.17	−0.24	0.11	C/**T**	TraesCS5B01G425500	Zinc finger CCCH domain-containing protein 16
		BLUEQ	3.14	−0.29	0.10			
S2B_717098540	NDVIa	BLUEC	8.41	−0.02	0.22	C/T	TraesCS2B01G522200	SAUR-like auxin-responsive protein family
		BLUEA	4.17	−0.01	0.18			
S1B_677572998	NDVIg	BLUEA	3.48	0.02	0.12	C/**G**	TraesCS1B01G467900	Methyltransferase
		BLUEQ	3.32	0.03	0.07			
S2D_570960728	NDVIg	BLUEQ	3.79	−0.03	0.07	A/**C**	TraesCS2D01G464800	Multidrug resistance protein ABC transporter family protein
		BLUEA	3.13	−0.02	0.13			
S3A_737114441	NDVIg	BLUEQ	3.49	0.02	0.07	T/**C**	TraesCS3A01G519100	rRNA N-glycosidase
		BLUEA	3.41	0.02	0.14			
S3B_785311773	NDVIg	BLUEQ	3.34	0.01	0.07	A/**G**	TraesCS3B01G550500	Myb/SANT-like DNA-binding domain protein
		BLUEA	3.01	0.01	0.13			
S5B_583295527	NDVIg	BLUEC	6.60	0.02	0.12	A/**G**	TraesCS5B01G407600	Myb family transcription factor-like protein
		BLUEA	5.00	0.02	0.16			
S7B_426882778	NDVIg	BLUEQ	3.28	0.03	0.08	A/**G**	TraesCS7B01G226400	RING finger protein 13
		BLUEA	3.01	0.02	0.14			
S3B_785311769	NDVIa	BLUEC	6.02	0.00	0.22	A/**G**	TraesCS3B01G550500	Myb/SANT-like DNA-binding domain protein
	NDVIg	BLUEQ	3.34	0.01	0.07			
	NDVIg	BLUEA	3.01	0.01	0.13			
S7B_701649275	NDVIa	BLUEA	5.82	0.00	0.20	C/**T**	TraesCS7B01G434600	FBD-associated F-box protein
	NDVIg	BLUEA	3.78	0.01	0.15			
S3A_699988530	NDVIg	BLUEQ	4.46	−0.04	0.12	A/**G**	TraesCS3A01G466000	F-box family protein
		BLUEA	3.48	−0.03	0.16			
S7D_635578722	NDVIg	BLUEC	4.17	0.02	0.11	T/**C**	TraesCS7D01G552700	NBS-LRR disease resistance protein
	NDVIa	BLUEA	3.49	0.01	0.18			
	SPAD	BLUEQ	3.41	−3.40	0.15			
	NDVIa	BLUEQ	3.34	0.02	0.09			
	NDVIg	BLUEA	3.15	0.03	0.15			

SPAD, soil-plant analyses development; MT, cell membrane thermostability; CT, canopy temperature (°C); NDVIa, normalized difference vegetation index at GS65; NDVIg, normalized difference vegetation index at grain filling. BLUEC, BLUEs values derived across Citra; BLUEQ, BLUEs values derived across Quincy, and BLUEA: BLUEs values derived across all environment; PVE, phenotypic variance explained

## Discussion

Growth and development of wheat is very sensitive to HT during anthesis and grain filling [7]. The impact of HDT and HNT on wheat growth and grain yield is well documented in many studies [7, 9, 21]. In this study, the SWAMP was evaluated in two heat prone environments of southeast USA with a goal to identify significant MTAs for use in breeding to improve adaptability and optimize yield potential. Short episodes of HDT (> 30 °C) and HNT (> 21 °C) were common during from anthesis to grain filling period in both environments (Additional file 1), thus, the MTAs identified in this study can provide useful information to understand the genetic bases of PTs under HDT and HNT.

**Fig. 1:**
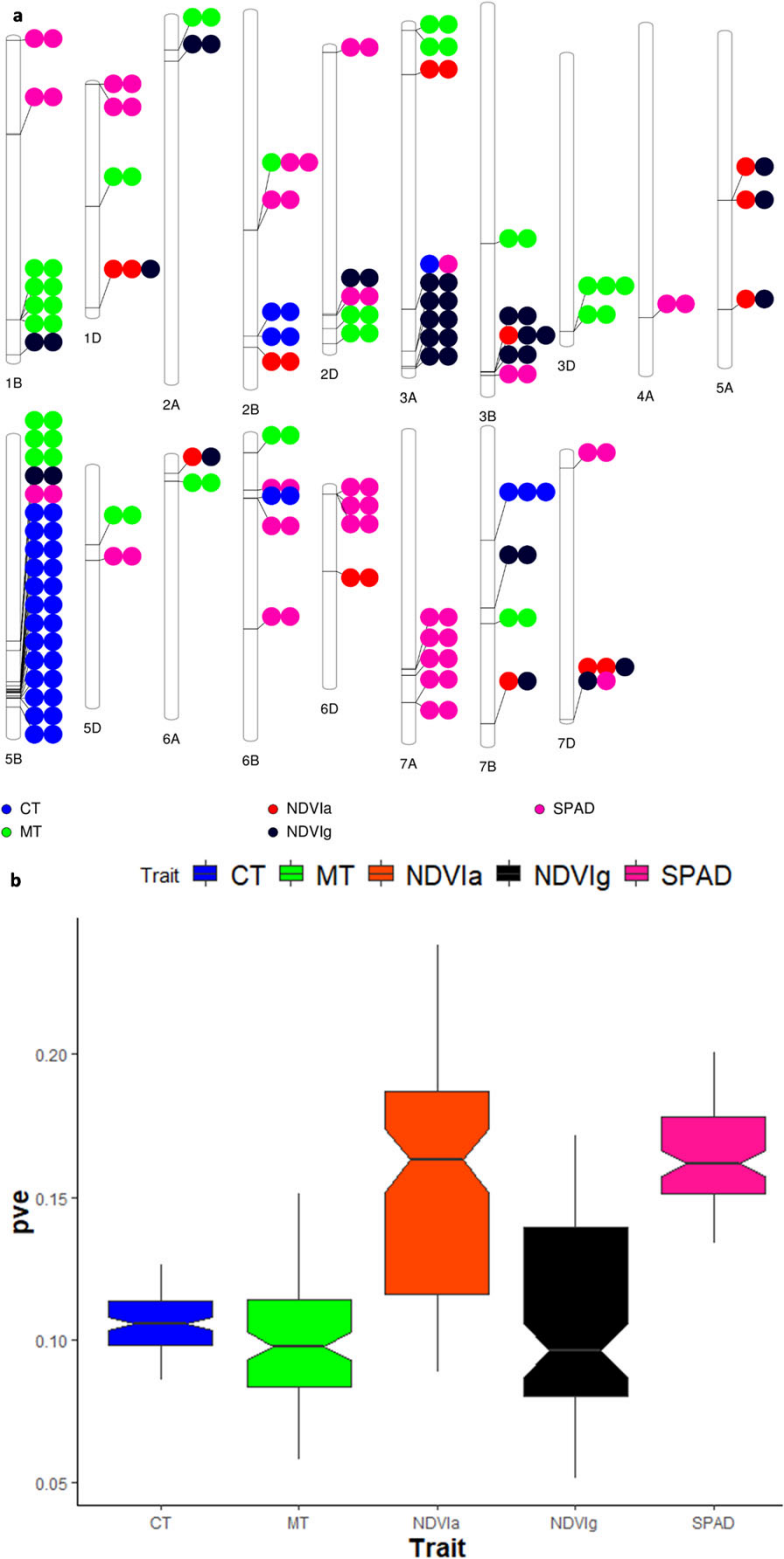
Summary of GWAS. **a** genome-wide distribution of stable markers trait associations and **b**) range of percentage of variation explained for physiological traits in SWAMP. SPAD, soil-plant analyses development; MT, cell membrane thermostability; CT, canopy temperature (°C); NDVIa, normalized difference vegetation index at GS65; NDVIg, normalized difference vegetation index at grain filling

HT reduces leaf area index and increases senescence rate which subsequently impairs photosynthesis and reduces grain yield [15, 22]. Measuring chlorophyll content using SPAD, as a proxy for the entire photosynthetic complex, indicates photosynthetic potential. Higher expression of SPAD values during reproductive stages in wheat have been associated with heat tolerance resulting in higher grain yield potential [23–25]. In this study, genotypes showed significant genotypic variation in SPAD values with moderate broad-sense heritability (0.49) (Table 1). Previous studies reported similar heritability for SPAD values [20]. Pearson’s correlation showed strong positive relationship of SPAD with GY, SF, GN, HI, SHI, TGW, and MT. The result was supported by PC biplot analysis where SPAD was clustered with GY, SF, GN, HI, SHI, and MT (Additional file 5). We identified 94 MTAs for SPAD with PVEs ranging from 13 to 20% (Table 2, Fig. 1b) out of which 24 MTAs were expressed in multiple environments suggesting the genetic stability of these MTAs under different environments (Table 4). Stable MTAs were located in chromosomes 1B, 1D, 2B, 2D, 3B, 4A, 5B, 5D, 6B, 6D, 7A, and 7D (Fig. 1a). In this study, SPAD shared common MTAs with MT (S2B_466014434), CT (S3A_609909640), and NDVIa and NDVIg (S7D_635578722) (Table 3). These are potential new targets for multi-trait improvement and marker assisted breeding. Thirteen of these stable MTAs had functional annotation suggesting their involvement in abiotic stress including heat stress (Table 5). Three markers within 9 bp range on chromosome 6D (S6D_16178496, S6D_16178499, S6D_16178505) were within gene, TraesCS6D01G038900, with functional annotation of 2-oxoglutarate (2OG) and Fe (II)-dependent oxygenase superfamily protein. This gene has been reported to increase oxidative stress and reduce flower and pod number in canola when affected by heat stress [26]. One MTA on chromosome 4A (S4A_625244392) within gene TraesCS4A01G346600 had functional annotation as F-box protein. A gene (TaFBA1) encoding homologous F-box protein was reported to regulate gene expression and improve enzymatic antioxidant levels in response to heat stress in tobacco [27]. These proteins area involved in regulating many other biological processes, including biotic and abiotic stresses, floral development, embryogenesis, hormonal responses, and senescence [28]. Another MTA (S2D_574118879) within gene TraesCS2D01G469000 (GDSL-like Lipase/Acylhydrolase superfamily protein) has a predicted functional role in response to thermal stress in sorghum [29]. We also found MTAs associated with drought stress. Moreover, we found several other MTAs whose annotations suggest different roles including response to drought stress (TBC1 domain family member, SNF1-related protein kinase regulatory subunit beta-2) plant senescence (E3 ubiquitin-protein ligase ORTHRUS 2), salt stress (Glutamyl-tRNA (Gln) amidotransferase subunit A), disease resistance (Disease resistance protein (NBS-LRR class) family), metabolism (Adenosine kinase-like protein) and antioxidant defense (Telomere repeat-binding factor like-protein) [30–36]. Our study identified novel MTAs associated with SPAD measurements in US soft wheat under HT conditions which contributes to a better understanding of the genetic basis of SPAD traits in wheat. Upon further validation, these MTAs can be used in future marker assisted breeding programs to overcome sink limitations, improve HI and ultimately increase yield potential.

Another important trait that provides rapid measurement of crops to characterize the canopy for LAI and leaf greenness is NDVI. High leaf chlorophyll content at anthesis and the ability to retain greenness (delayed senescence) during grain filling stages is associated with higher heat tolerance [37]. Studies have confirmed that NDVI could be used to predict grain yield in wheat [14]. We found significant genotypic variation among SWAMP genotypes for NDVI at anthesis and grain filling period. NDVIa and NDVIg showed moderate broad sense heritability of 0.56 and 0.40 respectively (Table 1) which aligned with the results from previous studies [38]. NDVI showed significant positive correlation with GY, GN, HI, TGW, and MT at anthesis and grain filling stages. Some studies have reported a strong correlation of NDVI with wheat grain yield at any growth stages [14]. Others have reported a varied relationship of NDVI with grain yield, depending on growth conditions [39]. Here, we found several unique as well as common MTAs associated with NDVIa and NDVIg. We identified total 102 MTAs for NDVIa with PVEs ranging from 8 to 23% (Table 2, Fig. 1b). Six out of 95 MTAs were expressed in several environments indicating stability of these markers under different environments (Table 4). For NDVIg, 99 significant MTAs were detected with PVE ranging from 5 to 17% (Table 2, Fig. 1b) with 14 stable MTAs (Table 2, Table 4, Fig. 1a). One MTA for NDVIa on chromosome 2B (S2B_717098540) is annotated as SAUR-like auxin-responsive protein family (TraesCS2B01G522200) and has been reported to be upregulated under heat stress in Arabidopsis [40]. For NDVIg, MTA on chromosome 1B (S1B_677572998) within gene TraesCS1B01G467900 is annotated as Methyltransferase which has a predicted role of genetic or epigenetic regulation of heat responses in plants [41]. Another MTA (S5B_583295527) within gene TraesCS5B01G407600 had functional annotation for Myb family transcription factor-like protein. Myb transcription factor were reported to be significantly induced by heat treatment in rice and wheat and thus play important roles in response to high temperature stress [42, 43]. Moreover, we found several other MTAs whose annotations suggest different roles including response to abiotic stress including drought stress (Multidrug resistance protein ABC transporter family protein, Myb/SANT-like DNA-binding domain protein, RING finger protein 13) [44–47]. Eight common MTAs were detected for NDVI at anthesis and grain filing period on chromosomes 1D, 3B, 5A, 6A, 7B, and 7D which can be potentially important targets for marker assisted selection. Three MTAs (S3A_699988530, S7B_701649275, and S7D_635578722) have annotations suggesting their role in heat tolerance [27, 28, 48]. Our study identified novel MTAs associated with NDVI which provides insight into the genetic basis of this trait in wheat at different growth stages in US soft wheat under HDT and HNT conditions.

The selective permeability of plasma membrane is highly sensitive to heat stress affecting growth and development of a plant [49]. Therefore, MT is another important PT for understanding heat tolerance in wheat where lower expression of solute leakage leaf tissue indicates stability (tolerance) of cell membrane to elevated temperature [24]. We measured solute leakage from heat stressed plant tissue to estimate damage to cell membrane [50]. Overall, we found significant genotypic variation in SWAMP for MT with moderate broad sense heritability (0.60) (Table 1) in agreement with previous studies [51]. MT was positively correlated with GY, SF, GN, HI, SHI, TGW, NDVIa, NDVIg and SPAD indicating that MT can be used as an additive component trait to improve yield potential in wheat under HT (Additional file 4). We identified 95 MTAs for MT with PVEs ranging from 5 to 15% (Table 2, Fig. 1b) indicating its quantitative nature. Twenty out of 95 MTAs were expressed in multiple environments indicating stability of these markers under different environments (Table 4). Twenty stable MTAs were located in chromosomes 1B, 1D, 2A, 2D, 3A, 3B, 3D, 5B, 5D, 6A, 6B, and 7B (Fig. 1a). Seven of these stable MTAs had functional annotation suggesting their involvement in abiotic stress including heat stress (Table 5). Two MTAs (S3D_590224603, and S3D_590224620) within 17 bp of each other were detected within gene TraesCS3D01G501200 with a functional annotation of protein kinase (Table 5). Protein kinases have been found to play a role in plant defense and adaptation responses and were reported to be upregulated by heat stress in durum wheat [52]. Another MTA (S1D_262475151) within gene TraesCS1D01G190700 (Heavy metal transport/detoxification superfamily protein) was reported to have an important role in growth and development of canola under heat stress conditions [53]. An MTA on chromosome 6B (S6B_42215716) within gene TraesCS6B01G063500 is annotated as peroxidase. Heat stress triggers the production and accumulation of harmful reactive oxygen species like hydrogen peroxide and their detoxification by antioxidant systems is important for protecting plants against heat stress [54, 55]. A significant increase in the activity of peroxidase (antioxidant) under short term heat stress has been reported in heat tolerant genotypes indicating efficient antioxidative defense system in wheat [56]. Two MTAs within 23 bp on chromosome 2B (S5B_509105168 and S5B_509105191) were in gene TraesCS5B01G325000 with annotation as F-box family protein.

Another parameter that has been frequently used to estimate heat tolerance in wheat is CT [57]. Lower canopy temperature indicates water status and transpiration rate in controlling temperature to avoid dehydration under stress [37, 58]. In this study, there was significant genotypic variation in CT with moderate broad-sense heritability (0.35) (Table 1). CT showed a significant negative correlation with GY, GN, SPAD, and MT. Genotypes with cooler canopies are presumed to have better root systems and retain chlorophyll content and membrane stability resulting in higher yield under high temperature. We identified 110 MTAs for CT with PVEs ranging from 8 to 13% (Table 2, Fig. 1b). Seventeen out of 110 MTAs were expressed in multiple environments indicating stability of these markers under different environments (Table 4). Stable MTAs were located in chromosomes 2B, 5B, 5D, 6B, and 7B (Fig. 1a). Ten of the stable MTAs were located in five genes with annotated functions. An MTA on chromosome 5B (S5B_610295429) is within gene TraesCS5B01G436700 which is annotated as a lipid transfer protein. Lipid transfer proteins are low molecular weight proteins that are involved in many biological roles, such as anther development, different signaling pathways and heat stress both at the seedling and the grain-filling stages [59]. One MTA (S5B_621237427) on chromosome 5B was found in gene (TraesCS5B01G448700). This gene is annotated as mitochondrial transcription termination factor-like protein and is reported to be involved in heat tolerance in Arabidopsis [60]. Another MTA detected on chromosomes 5B (S5B_601343966), has gene annotation for Zinc finger CCCH domain-containing protein 16 (TraesCS5B01G425500). This protein was found to be over expressed upon high temperature stress in bread wheat [61]. We found several other MTAs whose annotations suggest different roles including abiotic stress response (BTB/POZ and MATH domain-containing protein 2), senescence (Maintenance of telomere capping protein 2, Protein Phosphatase 2C) and salinity stress (Peptidase M50 family protein) [62–64].

**Fig. 2:**
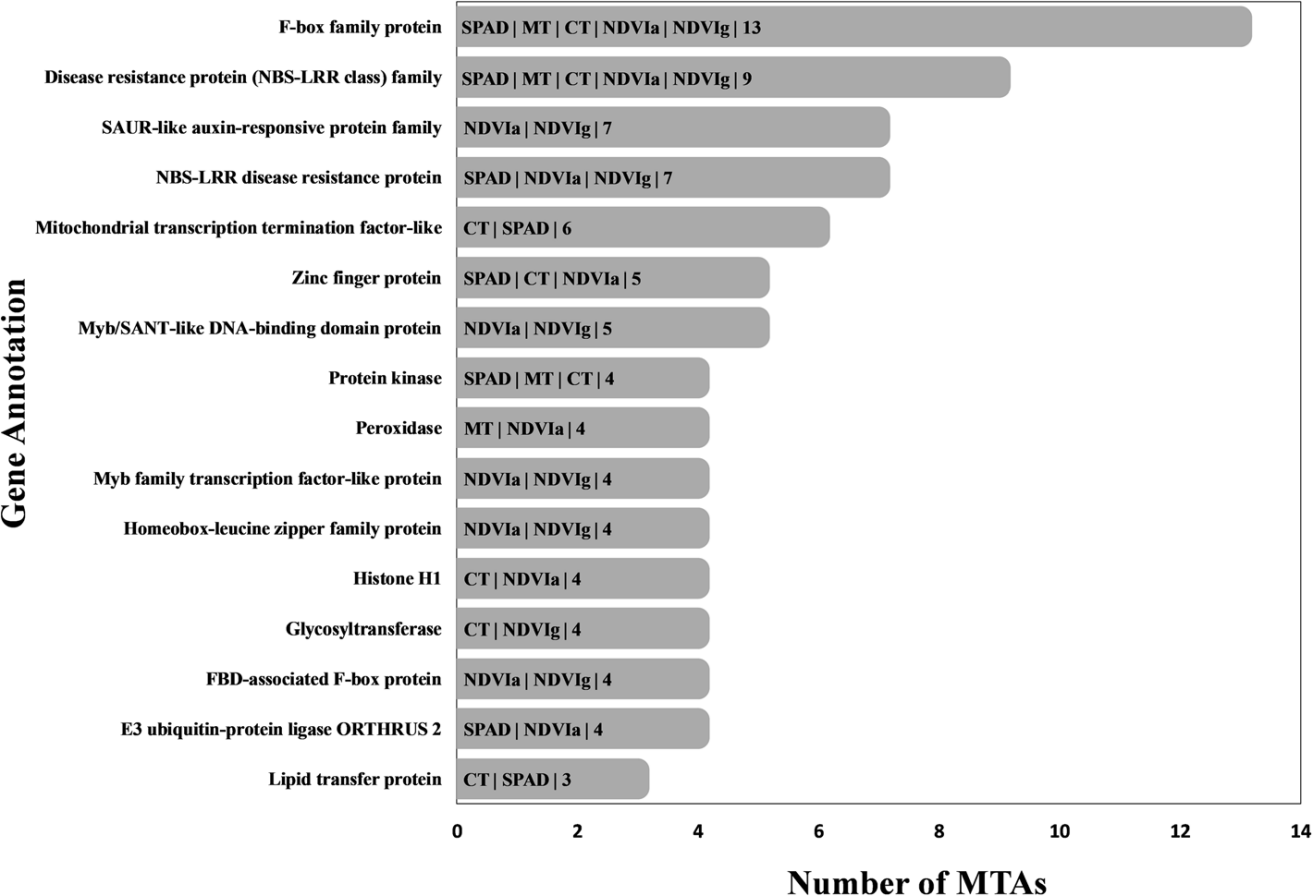
Potential candidate gene functions harboring SNPs affecting physiological traits under heat stress. The traits and count of marker–trait associations (for two or more traits) located within genes that have the same gene annotation is shown inside a bar. SPAD, soil-plant analyses development; MT, cell membrane thermostability; CT, canopy temperature (°C); NDVIa, normalized difference vegetation index at GS65; NDVIg, normalized difference vegetation index at grain filling

In summary, we detected 500 MTAs located in different chromosomes out of which 81 MTAs were linked to the same trait in multiple environments (suggesting stability) and ten MTAs linked to multiple traits (suggesting pleiotropy) (Table 3-4). Notably, MTAs associated with multiple PTs within different genomic regions had the same functional annotation (Fig. 2; and Additional file 8). For instance, 13 MTAs for SPAD, MT, CT, NDVIa and NDVIg were annotated as F-box family proteins (Fig. 2). Similarly, the genes annotated as zinc finger proteins harbored MTAs for SPAD, CT and NDVIa. This result indicated the likely gene families that are important for physiological traits to improve yield potential under heat stress.

Some MTAs associated with PTs had pleiotropic effects with GY and other yield related traits (Additional file 9). These MTAs have been described in our previous studies. In a previous study [10], we found two MTAs (S3A_12554694 and S3A_12554700) within 6 bp were associated with TGW and GY. In this study, we found the same MTA associated with MT indicating that plasma membrane thermostability may contribute to increased TGW and GY under heat stress condition. Another MTA on chromosome 7D (S7D_18808932) also had a pleiotropic effect on MT and TGW. MTAs with pleiotropic effect on CT and TGW were detected on chromosome 6B (S6B_149148874) under multiple HT environments. One MTA (S5A_590056740), associated with HI in our previous study [10] was also linked to NDVIa and NDVIg. The co-localization of observed MTAs for HI with NDVIa and NDVIg suggests that HI is highly dependent upon greenness at anthesis as well as grain filling period under heat stress. This might indicate that higher photosynthetic reserves at anthesis can later be translocated to the developing grain.

In recent years, rapid progress in ground-based and unmanned aerial high-throughput field phenotyping have resulted in a variety of non-invasive imaging techniques which can lead to significant improvements in precision and speed of phenotyping for PTs in large plant populations with high resolution and high precision. These platforms can have large impact on validating as well as finding new genetic loci that are relevant to heat tolerance.

## Conclusion

A large number of MTAs were identified in soft red winter wheat under the environments with HDT and HNT conditions. The MTAs were detected for four PTs: SPAD, MT, CT, NDVI for which there is reasonable evidence of being heat adaptive. We found several stable loci across environments and pleiotropic markers controlling multiple traits among PTs and also associated with yield-related traits under hot environments. We identified candidate genes affecting several important biological processes in plants including response to heat stress. Identifying regulatory loci associated with traits like PTs and yield-related traits can assist in developing ideotypes that can maximize the amount of assimilates and conversion of enhanced carbon capture and biomass growth for improving yield potential. Further validation of these MTAs in different controlled environmental conditions is required before they can be used extensively in marker assisted selection and breeding for heat tolerance in wheat.

## Methods

### Plant materials and experimental design

Field evaluations were conducted on 236 advanced genotypes of a soft red winter wheat association mapping panel (SWAMP) that are well adapted to the warm and humid south and southeastern regions of the USA. These lines were developed by public and private soft wheat breeding programs in the south and southeastern USA and the list is available in the NCBI database with accession number PRJNA578088 (https://www.ncbi.nlm.nih.gov//bioproject/PRJNA578088). The seeds were collected from different soft wheat breeding programs (University of Arkansas, University of Georgia and Louisiana State University) from in the south and southeastern USA. The SWAMP was evaluated for PTs in five trials at the two heat stressed locations in Florida: Citra and Quincy. Citra had more frequent episodes of HT (> 30 °C) during the grain filling period than Quincy (Additional file 1). In Citra, the SWAMP was phenotyped for three growing seasons: 2015–2016 (29.407215 °N, − 82.1876 °W, Elevation = 23 m), 2016–2017 (29.405701 °N, − 82.175818 °W, Elevation = 23 m), and 2017–2018 (29.403853 °N, − 82.17429 °W, Elevation = 23 m). In Quincy, SWAMP was evaluated in two growing seasons: 2015–2016 (30.5546202 °N, − 84.59533 °W, Elevation = 76 m) and 2016–2017 (30.549658 °N, − 84.59835 °W, Elevation = 76 m). Traits assessed in each year and used in GWAS can be found in (Additional file 2). All yield trials were planted in six row plots (3 m length × 1.5 m width) at the seeding rate of 100 kgh^− 1^. The SWAMP was planted in randomized augmented block design [65] in all trials with 236 un-replicated entries and three repeated check varieties (SS8641, AGS2000 and Jamestown).

### Trait measurement

Four physiological traits (PTs) including CT, SPAD, NDVI, and MT were measured at different time points. CT was measured three times during grain filling period (Zadoks stages 67, 72, and 77) using a handheld infrared thermometer (Fluke 572–2 IR thermometer, Fluke Corporation, Everett WA) on sunny days when the temperature reached the daily high between 1300 and 1500 h [66]. CT data were taken from the same side of each plot at 50 distance from the edge and approximately 50 cm above the canopy at an angle of 30^o^ to the horizontal. The average of three time point readings was used for the association analysis. Chlorophyll content was measured on flag leaves from eight random main stems for each plot at anthesis plus seven days (Zadoks stage 72) using a handheld self-calibrated SPAD chlorophyll meter (Minolta SPAD-502 Spectrum Technologies Inc., Plainfield, IL, US). The SPAD-502 instrument provides a convenient means of assessing relative leaf chlorophyll concentration. The chlorophyll content was measured on intact flag leaves one third of the way from the base of the abaxial surface. The average of eight readings was used for further statistical analysis.

SPAD Chlorophyll meter data were taken on the same day or the closest possible day coinciding with CT and MT. NDVI was measured at anthesis (NDVIa; Zadoks stage 65) and grain filling stage (NDVIg; Zadoks stage 77). A GreenSeeker handheld crop sensor (Trimble Navigation Limited 935 Stewart Drive Sunnyvale, California 94,085) was used for collecting NDVI readings. The GreenSeeker handheld crop sensor was hold 50 cm above the canopy facing the center of the bed. A 30–40 NDVI readings were recorded/plot and the mean value of those readings represented the NDVI value of the respective plot. To determine MT, flag leaves were collected from ten random main stems at anthesis plus seven days (Zadoks stage 72) from each plot. One cm diameter leaf disks from each leaf were extracted from midway between the base and the tip of the leaf blade using a leaf disc puncher and placed in glass vials containing 20 ml deionized water. The vials were placed in shaker for 24 h at room temperature to ensure diffusion of electrolytes. After 24 h, electrolyte leakage was measured using a conductometer (Thermo Scientific Orion Star A212) followed by autoclaving the vials (0.10 MPa pressure, 121 °C for 15 min) to release all the electrolytes from plant tissue. The vials were placed in shaker for 24 h and electrolyte leakage was measured again. MT was expressed in percentage units as the reciprocal of relative leakage [50].

$$ \mathrm{MT}=\left(1-{\mathrm{T}}_1/{\mathrm{T}}_2\right)\ \mathrm{x}\ 100 $$

where T_1_ is the conductivity reading after heat treatment, and T_2_ is the conductivity reading after autoclaving. Grain yield (GY) and yield-related traits including grain number (GN), harvest index (HI), thousand grain weight (TGW), spike fertility (SF), and spike harvest index (SHI) were also calculated to determine correlation among traits. The details of the calculation of these traits is described in previous study [10].

### Phenotypic data analysis

Combined analysis of variance (ANOVA) was conducted assuming a mixed linear model. The ‘lme4’ package [67] and the R software program (v3.5.1, R Development Core Team) were used to calculate best linear unbiased estimates (BLUEs) assuming a fixed genotypic effect (all other effects were random):

$$ {Y}_{ij k}=\mu +{G}_i+{E}_j+{GE}_{ij}+{B}_k{(E)}_j+{\varepsilon}_{ij k} $$

where the phenotypic response (Y_ijk_) is a function of the overall mean (μ), *i*th genotype (G_i_), *j*th environment, genotype-environment interaction (GE_ij_), *k*th block (B_k_) nested within the *j*th environment (E_j_), and the residual error (ε_ijk_).

BLUEs for combined as well as individual locations were also calculated and therefore will be discussed hereafter as BLUEC (BLUE values estimated from Citra), BLUEQ (BLUE values estimated from Quincy) and BLUEA (BLUE values estimated from all environments). Broad sense heritability was calculated assuming random genotypic effect (all other effects were random) and was obtained by:

$$ {H}^2=\frac{\sigma_G^2}{\sigma_G^2+{\sigma}_{\frac{GxE}{n}}^2+{\sigma}_{\frac{e}{nr}}^2} $$

where *H*^*2*^, broad-sense heritability estimate; σ^2^_G_, genetic variance; σ^2^_G × E_, genotype-by-environmental variance; σ^2^_e,_ residual variance; n, number of environments; and r, number of replications.

Pearson’s correlations were calculated from BLUEs in R using the “corrplot” package (v3.5.1, R Development Core Team) and used to determine the direction and magnitude of measured trait associations. Associations between traits were also explored in principle component (PC) biplot analysis using the package “factoextra” in R [68].

### Genotyping

The detail of genotyping process, SNP discovery and filtering criteria has been described in previous study [10]. In brief, DNA was isolated from fresh, young leaves using a modified Cetyl trimethylammonium bromide (CTAB) protocol [69]. The GBS libraries were prepared using MspI and PstI-HF restriction enzymes and pooled together in 96-plex and sequenced in an Ion Torrent Proton sequencer (Thermo Fisher Scientific, Waltham, MA, USA) at the USDA Central Small Grain Genotyping Lab, Kansas State University, Manhattan, KS, USA. Prior to analysis, 80 poly-A bases were appended to the 3′ end of all sequencing reads so that TASSEL 5.0 would attempt to use reads shorter than 64 bases rather than discarding short reads. SNP calling was performed in TASSEL v5.0 GBS v2.0 discovery pipeline [70] and aligned to the Chinese Spring wheat (RefSeq v1) genome sequence [71] using the default settings of BWA (version 0.6.1). The markers were filtered based on the criteria of minor allele frequency (MAF > 5%) and missing values (< 20%)**.**

### Linkage disequilibrium, population structure and GWAS analysis

Linkage disequilibrium (LD) and population structure analysis of the SWAMP has been described in detail previously [10]. Briefly, “LDcorSV” package [72] in R (v3.5.1, R Development Core Team) was used to estimate LOESS (Locally weighted scatterplot smoothing) regressions of mean r^2^ (coefficient of LD) between pairs of SNPs sampled at the range of 30,000, 40,000, and 50,000 bp. The intersection between critical value (r^2^ = 0.2) and LOESS line was considered as the distance beyond which LD starts to decay. Population structure was observed using Bayesian information criterion (BIC) score provided by discriminant analysis of principal components (DAPC, “adegenet” package, R Development Core Team 2013) [73] to determine the optimum number of demes supported by the results. The principal component analysis was performed using “prcomp” (“stats” package) to investigate the genetic differentiation among and within demes.

GWAS was performed in three BLUE datasets (BLUEC, BLUEQ, BLUPA) for each trait to identify significant MTAs in SWAMP using Fixed and random model Circulating Probability Unification (FarmCPU) model [74, 75] executed in the Genome Association Prediction Integrated Tool (GAPIT) package in R software package [76]. The first three principal components were used as covariates by observing model fit in Q-Q (quantile-quantile) plots, and kinship was determined from FarmCPU [74]. A uniform value of -log10(*p)* = 4.00 (*p* = 9.99 × 10^− 4^) was used as the cut-off to define significant MTAs based on Q-Q plots [77, 78]. Candidate genes associated with significant MTAs and their annotation were identified using Chinese Spring wheat reference genome (IWGSC RefSeq v1.0) [71]. The putative genes were further investigated in past literature to determine their association with phenotypic traits under heat stress.

## Supplementary information

**Additional file 1.** Weather table showing number of hours in daytime (> 24 °C) and nighttime (> 15 °C) temperature during grain filling stages (Mar 15 - Apr 30). The soft wheat association mapping panel (SWAMP) was planted in three seasons in Citra (2015/2016, 2016/2017, 2017, 2018) and two seasons in Quincy (2015/2016, 2016/2017).

**Additional file 2.** Physiological traits for the SWAMP assessed in each year and used in GWAS.

**Additional file 3.** Summary of ANOVA results testing the effects of genotype (G), environment (E), and genotype-by-environment interaction (G × E). The table includes mean square values and significance level of each term. SPAD, soil-plant analyses development; MT, cell membrane thermostability; CT, canopy temperature (°C); NDVIa, normalized difference vegetation index at GS65; NDVIg, normalized difference vegetation index at grain filling.

**Additional file 4.** Pearson’s correlation coefficient (r) between physiological traits in SWAMP. SF, spike fertility (grains g-1 chaff weight); GY, grain yield (kg h-1); GN, grain number m-^2^; TGW, thousand grain weight (g); SHI, spike harvest index; HI, harvest index; SPAD, soil-plant analyses development; MT, cell membrane thermostability; CT, canopy temperature (°C); NDVIa, normalized difference vegetation index at GS65; NDVIg, normalized difference vegetation index at grain filling.

**Additional file 5.** Principal component bi-plot analysis of measured traits for the SWAMP. SF, spike fertility (grains g^− 1^ chaff weight); GY, grain yield (kg h^− 1^); GN, grain number m^− 2^; TGW, thousand grain weight (g); SHI, spike harvest index; HI, harvest index; SPAD, soil-plant analyses development; MT, cell membrane thermostability; CT, canopy temperature (°C); NDVIa, normalized difference vegetation index at GS65; NDVIg, normalized difference vegetation index at grain filling.

**Additional file 6.** Population structure of the SWAMP based on 27,466 SNPs. (A) bar charts showing posterior probabilities of assignment to three groups based on algorithms of discriminant analysis of principal components (DAPC). (B) Population structure among demes inferred from PC analysis. The populations were colored based on the posterior of probability assigned to three genetic groups inferred from DAPC.

**Additional file 7.** Linkage disequilibrium represented by the r^2^ against physical distance (in bp) showing LD decay. LOESS regressions of mean r2 between pairs of SNPs vs. physical distance were sampled at 30,000 (red), 40,000 (blue), and 50,000 (green) bp. Grey line represents the critical value beyond which LD is likely caused by physical linkage.

**Additional file 8.** Summary of all significant markers and their functional annotations associated with eight traits in SWAMP.

**Additional file 9.** Summary of significant pleiotropic MTAs associated with PTs, GY and other yield related traits.

## Data Availability

The phenotypic datasets used and/or analyzed during the current study are available from the corresponding author on reasonable request. The genotypic datasets generated and/or analyzed during the current study are available in the NCBI using accession number PRJNA578088 (https://www.ncbi.nlm.nih.gov//bioproject/PRJNA578088).

## Abbreviations

HT: high temperature
MTAs: GWAS: genome-wide association study
MTAs: marker-trait associations
HDT: daytime maximum temperature
HNT: night-time minimum temperatures
HI: harvest index
PTs: physiological traits
RUE: radiation use efficiency
LI: light interception
SPAD: soil-plant analyses development
MT: membrane thermostability
CT: canopy temperature
NDVI: normalized difference vegetation index
NDVIa: normalized difference vegetation index at anthesis
NDVIg: normalized difference vegetation index at grain filling
QTL: quantitative trait loci
SWAMP: soft winter wheat association mapping panel
GY: grain yield
TGW: thousand grain weight
SF: spike fertility
SHI: spike harvest index
BLUEs: best linear unbiased estimates
BLUEC: BLUE values estimated from Citra
BLUEQ: BLUE values estimated from Quincy
BLUEA: BLUE values estimated from all environments
LD: linkage disequilibrium
FarmCPU: Fixed and random model Circulating Probability Unification
PVE: phenotypic variance explained
