# Supplementary material for: Genetic dissection of heat-responsive physiological traits to improve adaptation and increase yield potential in soft winter wheat
Source: BMC Genomics. 2020 Apr 20;21:315. doi: 10.1186/s12864-020-6717-7 (PMC7171738; doi:10.1186/s12864-020-6717-7)
Supplement: Supplementary file 1 — Additional file 1. Weather table showing number of hours in daytime (> 24 °C) and nighttime (> 15 °C) temperature during grain filling stages (Mar 15 - Apr 30). The soft wheat association mapping panel (SWAMP) was planted in three seasons in Citra (2015/2016, 2016/2017, 2017, 2018) and two seasons in Quincy (2015/2016, 2016/2017). [file 12864_2020_6717_MOESM1_ESM.docx]

**Additional file 1** Weather table showing number of hours in daytime (>24˚C) and nighttime (>15˚C) temperature during grain filling stages (Mar 15 - Apr 30). The soft Wheat association mapping panel (SWAMP) was planted in three seasons in Citra (2015/2016, 2016/2017, 2017, 2018) and two seasons in Quincy (2015/2016, 2016/2017).

|  | **Number of hours (˚C)** | | | | | |
| --- | --- | --- | --- | --- | --- | --- |
|  | **Day** | | | **Night** | | |
|  | **24-27** | **27-30** | **>30** | **15-18** | **18-21** | **>21** |
| **C16** | 190 | 134 | 56 | 101 | 226 | 115 |
| **C17** | 142 | 142 | 118 | 127 | 110 | 117 |
| **C18** | 148 | 127 | 15 | 225 | 122 | 48 |
| **Q16** | 132 | 55 | 10 | 177 | 130 | 44 |
| **Q17** | 168 | 174 | 9 | 174 | 110 | 69 |

# C16, Citra 2015/2016; C17, Citra 2016/2017; C18, Citra 2017/2018) Q16, Quincy 2015/2016; Q17, Quincy 2016/2017.
