# Supplementary material for: Genetic dissection of heat-responsive physiological traits to improve adaptation and increase yield potential in soft winter wheat
Source: BMC Genomics. 2020 Apr 20;21:315. doi: 10.1186/s12864-020-6717-7 (PMC7171738; doi:10.1186/s12864-020-6717-7)
Supplement: Supplementary file 2 — Additional file 2. Physiological traits for the SWAMP assessed in each year and used in GWAS. [file 12864_2020_6717_MOESM2_ESM.docx]

# Additional file 2: Physiological traits for the SWAMP assessed in each year and used in GWAS.

| Environment | SPAD | MT | CT | NDVIa | NDVIg |
| --- | --- | --- | --- | --- | --- |
| C16 | ✓ |  | ✓ | ✓ | ✓ |
| C17 | ✓ | ✓ | ✓ | ✓ | ✓ |
| C18 |  | ✓ | ✓ | ✓ | ✓ |
| Q16 |  |  | ✓ | ✓ | ✓ |
| Q17 | ✓ | ✓ | ✓ | ✓ | ✓ |

# C16, Citra 2015/2016; C17, Citra 2016/2017; C18, Citra 2017/2018) Q16, Quincy 2015/2016; Q17, Quincy 2016/2017.
