# Supplementary material for: Genetic dissection of heat-responsive physiological traits to improve adaptation and increase yield potential in soft winter wheat
Source: BMC Genomics. 2020 Apr 20;21:315. doi: 10.1186/s12864-020-6717-7 (PMC7171738; doi:10.1186/s12864-020-6717-7)
Supplement: Supplementary file 3 — Additional file 3. Summary of ANOVA results testing the effects of genotype (G), environment (E), and genotype-by-environment interaction (G × E). The table includes mean square values and significance level of each term. SPAD, soil-plant analyses development; MT, cell membrane thermostability; CT, canopy temperature (°C); NDVIa, normalized difference vegetation index at GS65; NDVIg, normalized difference vegetation index at grain filling. [file 12864_2020_6717_MOESM3_ESM.docx]

**Additional file 3:** Summary of ANOVA results testing the effects of genotype (G), environment (E), and genotype-by-environment interaction (G×E). The table includes mean square values and significance level of each term.

| **Trait** | **Genotype G** | **Environment E** | **Interaction GxE** |
| --- | --- | --- | --- |
| **SPAD** | 42.00*** | 573.20* | 22.80* |
| **MT** | 293.20*** | 94.00 | 132.50** |
| **CT** | 1.08*** | 834.84*** | 0.73* |
| **NDVIa** | 0.005*** | 0.03*** | 0.003*** |
| **NDVIg** | 0.01*** | 0.09*** | 0.007*** |

SPAD, soil-plant analyses development; MT, cell membrane thermostability; CT, canopy temperature (˚C); NDVIa, normalized difference vegetation index at GS65; NDVIg, normalized difference vegetation index at grain filling.
