# Supplementary material for: Genetic dissection of heat-responsive physiological traits to improve adaptation and increase yield potential in soft winter wheat
Source: BMC Genomics. 2020 Apr 20;21:315. doi: 10.1186/s12864-020-6717-7 (PMC7171738; doi:10.1186/s12864-020-6717-7)
Supplement: Supplementary file 4 — Additional file 4. Pearson’s correlation coefficient (r) between physiological traits in SWAMP. SF, spike fertility (grains g-1 chaff weight); GY, grain yield (kg h-1); GN, grain number m-2; TGW, thousand grain weight (g); SHI, spike harvest index; HI, harvest index; SPAD, soil-plant analyses development; MT, cell membrane thermostability; CT, canopy temperature (°C); NDVIa, normalized difference vegetation index at GS65; NDVIg, normalized difference vegetation index at grain filling. [file 12864_2020_6717_MOESM4_ESM.docx]

**Additional file 4**: Pearson’s correlation coefficient (r) between physiological traits in SWAMP.

| Traits | MT | CT | NDVIa | NDVIg | GY | SF | GN | HI | SHI | TGW |
| --- | --- | --- | --- | --- | --- | --- | --- | --- | --- | --- |
| SPAD | 0.31*** | -0.25*** | -0.01 | -0.04 | 0.50*** | 0.25*** | 0.30*** | 0.46*** | 0.37*** | 0.26*** |
| MT | 1 | -0.09 | 0.22*** | 0.31*** | 0.60*** | 0.29*** | 0.33*** | 0.58*** | 0.44*** | 0.40*** |
| CT | -0.25*** | 1 | 0.2** | 0.09 | -0.18** | -0.03 | -0.17* | 0.07 | -0.09 | -0.02 |
| NDVIa | 0.22*** | 0.2** | 1 | 0.78*** | 0.33*** | 0.04 | 0.17** | 0.49*** | 0.04 | 0.24*** |
| NDVIg | 0.31*** | 0.09 | 0.78*** | 1 | 0.40*** | 0.08 | 0.18** | 0.47*** | 0.16** | 0.31*** |

# SF, spike fertility (grains g-1 chaff weight); GY, grain yield (kg h-1); GN, grain number m-²; TGW, thousand grain weight (g); SHI, spike harvest index; HI, harvest index; SPAD, soil-plant analyses development; MT, cell membrane thermostability; CT, canopy temperature (˚C); NDVIa, normalized difference vegetation index at GS65; NDVIg, normalized difference vegetation index at grain filling.
