# Supplementary material for: Genetic dissection of heat-responsive physiological traits to improve adaptation and increase yield potential in soft winter wheat
Source: BMC Genomics. 2020 Apr 20;21:315. doi: 10.1186/s12864-020-6717-7 (PMC7171738; doi:10.1186/s12864-020-6717-7)

# Additional file 5: Principal component bi-plot analysis of physiological and agronomic traits for the SWAMP.

SF, spike fertility (grains g^-1^ chaff weight); GY, grain yield (kg h^-1^); GN, grain number m^-²^; TGW, thousand grain weight (g); SHI, spike harvest index; HI, harvest index; SPAD, soil-plant analyses development; MT, cell membrane thermostability; CT, canopy temperature (˚C); NDVIa, normalized difference vegetation index at GS65; NDVIg, normalized difference vegetation index at grain filling.


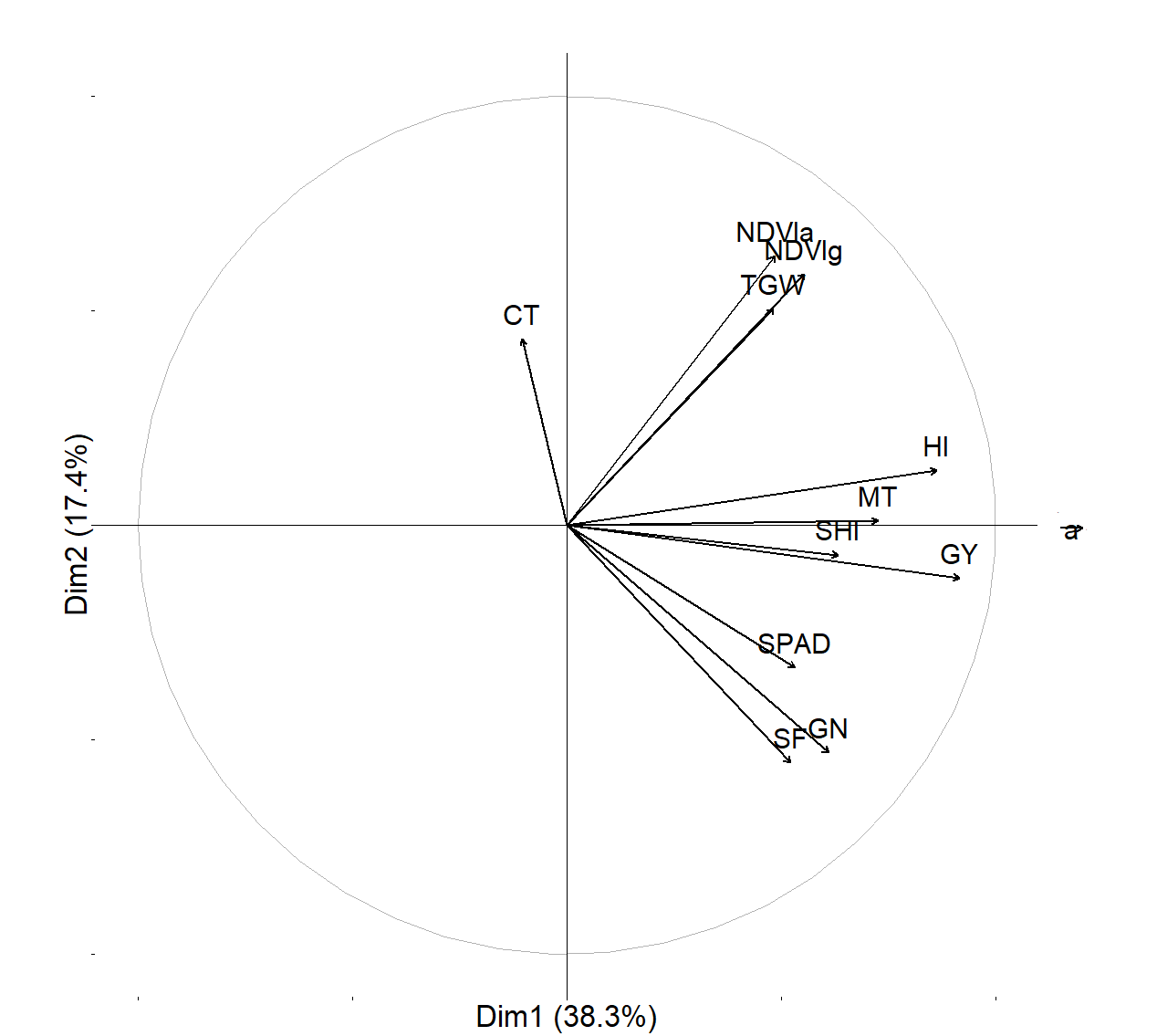

Supplement: Supplementary file 5 — Additional file 5. Principal component bi-plot analysis of measured traits for the SWAMP. SF, spike fertility (grains g− 1 chaff weight); GY, grain yield (kg h− 1); GN, grain number m− 2; TGW, thousand grain weight (g); SHI, spike harvest index; HI, harvest index; SPAD, soil-plant analyses development; MT, cell membrane thermostability; CT, canopy temperature (°C); NDVIa, normalized difference vegetation index at GS65; NDVIg, normalized difference vegetation index at grain filling. [file 12864_2020_6717_MOESM5_ESM.docx]
