# Supplementary material for: Genetic dissection of heat-responsive physiological traits to improve adaptation and increase yield potential in soft winter wheat
Source: BMC Genomics. 2020 Apr 20;21:315. doi: 10.1186/s12864-020-6717-7 (PMC7171738; doi:10.1186/s12864-020-6717-7)

**Additional file 6:** Population structure of the SWAMP based on 27,466 SNPs. (A) bar charts showing posterior probabilities of assignment to three groups based on algorithms of discriminant analysis of principal components (DAPC). (B) Population structure among demes inferred from PC analysis. The populations were colored based on the posterior of probability assigned to three genetic groups inferred from DAPC.

#
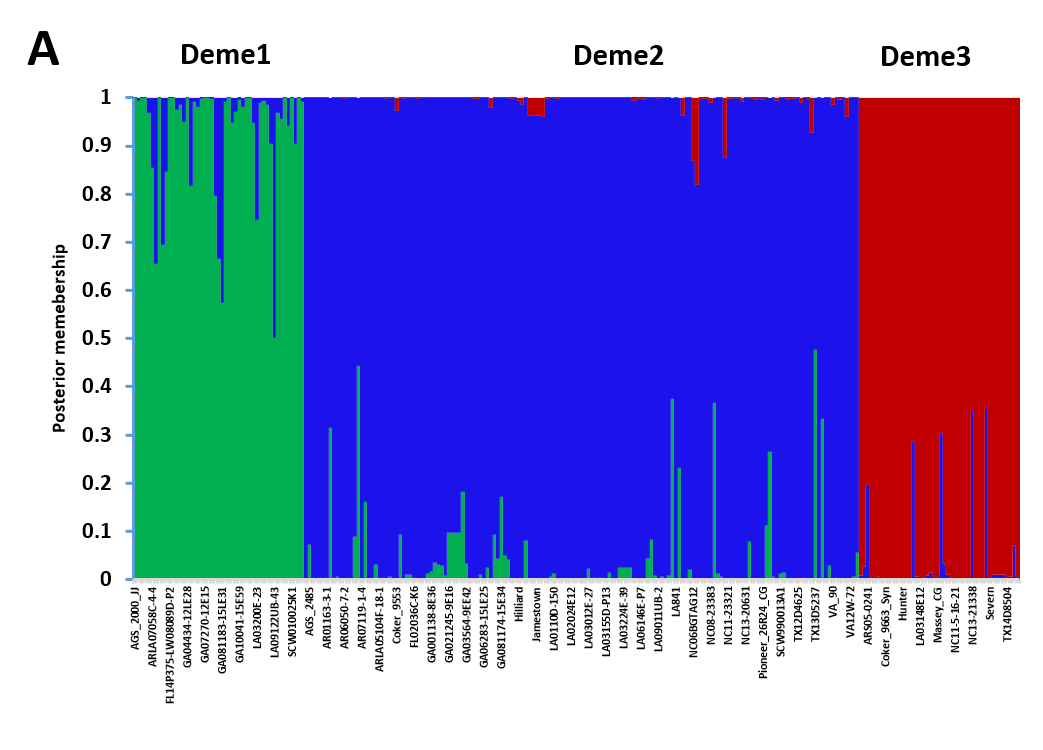


#
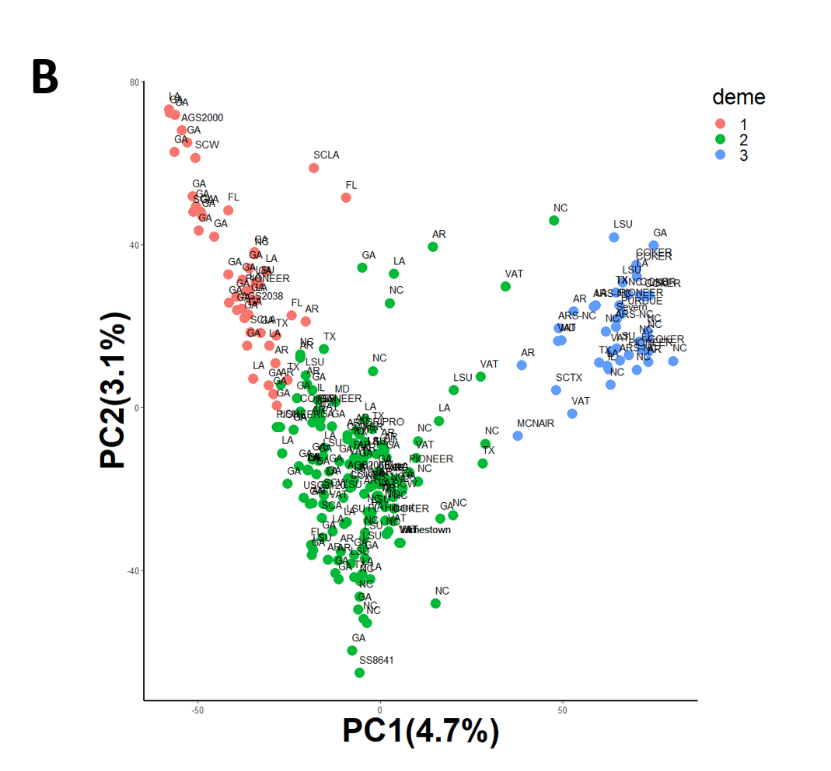

Supplement: Supplementary file 6 — Additional file 6. Population structure of the SWAMP based on 27,466 SNPs. (A) bar charts showing posterior probabilities of assignment to three groups based on algorithms of discriminant analysis of principal components (DAPC). (B) Population structure among demes inferred from PC analysis. The populations were colored based on the posterior of probability assigned to three genetic groups inferred from DAPC. [file 12864_2020_6717_MOESM6_ESM.docx]
