# Supplementary material for: Genetic dissection of heat-responsive physiological traits to improve adaptation and increase yield potential in soft winter wheat
Source: BMC Genomics. 2020 Apr 20;21:315. doi: 10.1186/s12864-020-6717-7 (PMC7171738; doi:10.1186/s12864-020-6717-7)

**Additional file 7**: Linkage disequilibrium represented by the r^2^ against physical distance (in bp) showing LD decay. LOESS regressions of mean r2 between pairs of SNPs vs. physical distance were sampled at 30,000 (red), 40,000 (blue), and 50,000 (green) bp. Grey line represents the critical value beyond which LD is likely caused by physical linkage.

#
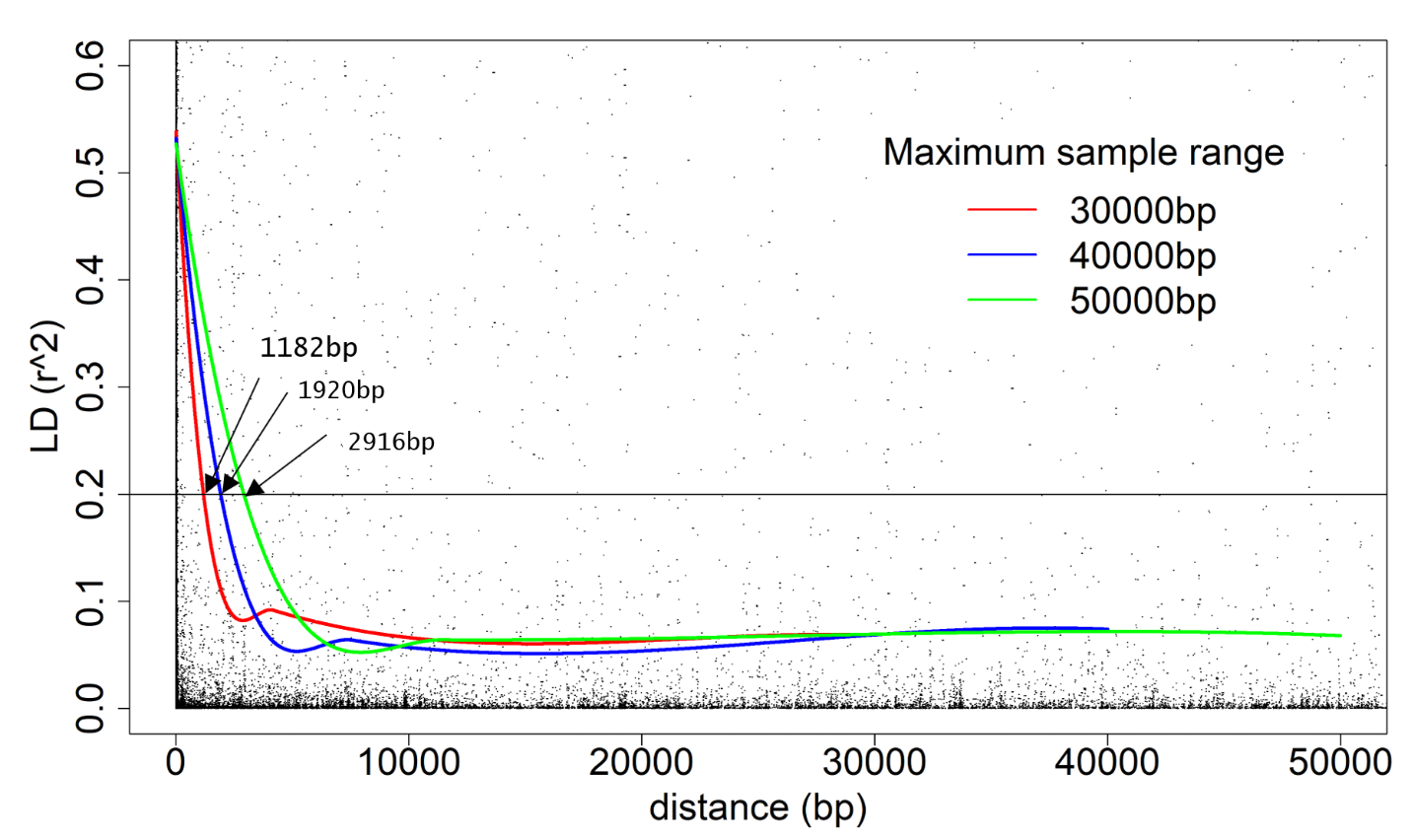

Supplement: Supplementary file 7 — Additional file 7. Linkage disequilibrium represented by the r2 against physical distance (in bp) showing LD decay. LOESS regressions of mean r2 between pairs of SNPs vs. physical distance were sampled at 30,000 (red), 40,000 (blue), and 50,000 (green) bp. Grey line represents the critical value beyond which LD is likely caused by physical linkage. [file 12864_2020_6717_MOESM7_ESM.docx]
